# *Limosilactobacillus vaginalis* Exerts Bifidogenic Effects: A Novel Postbiotic Strategy for Infant Prebiotic Supplementation

**DOI:** 10.3390/nu15204433

**Published:** 2023-10-19

**Authors:** Barbara Giordani, Carola Parolin, Angela Abruzzo, Claudio Foschi, Antonella Marangoni, Barbara Luppi, Beatrice Vitali

**Affiliations:** 1 Department of Pharmacy and Biotechnology, Alma Mater Studiorum, University of Bologna, 40126 Bologna, Italy; barbara.giordani4@unibo.it (B.G.); angela.abruzzo2@unibo.it (A.A.); barbara.luppi@unibo.it (B.L.); b.vitali@unibo.it (B.V.); 2 Section of Microbiology, Department of Medical and Surgical Sciences, University of Bologna, 40138 Bologna, Italy; claudio.foschi2@unibo.it (C.F.); antonella.marangoni@unibo.it (A.M.); 3 Microbiology Unit, IRCCS Azienda Ospedaliero-Universitaria di Bologna, 40138 Bologna, Italy

**Keywords:** *Limosilactobacillus vaginalis*, bifidobacteria, postbiotic, heat-killed probiotic, infant gut microbiota

## Abstract

Infant microbiota shaping strictly influences newborns’ well-being and long-term health, and babies born by cesarean-section and formula-fed generally show low microbial gut diversity and are more prone to develop various disorders. The supplementation with beneficial microbes of vaginal origin or derivatives (postbiotics, including heat-inactivated cells) represents a valid strategy to drive the correct gut microbiota shaping. Here, we explored for the first time the bifidogenic activity of a heat-killed vaginal strain (*Limosilactobacillus vaginalis* BC17), in addition to the assessment of its safety. *L. vaginalis* BC17 whole genome was sequenced by Nanopore technology and highlighted the absence of antibiotic resistance genes and virulence factors, indicating the strain safety profile for human health. MIC values confirmed that *L. vaginalis* BC17 is susceptible to widely employed antibiotics. Heat-killed BC17 cells significantly enhanced the planktonic growth of *Bifidobacterium* spp. For the first time, stimulating effects were observed also toward biofilm formation of bifidobacteria and their pre-formed biofilms. Conversely, heat-killed BC17 cells exerted antibacterial and anti-biofilms activities against Gram-positive and Gram-negative pathogens. Lyophilized heat-killed BC17 cells were formulated in a sunflower oil suspension (10^10^ heat-killed cell/g) intended for infant oral intake. This possessed optimal technological (i.e., re-dispersibility and stability) and functional properties (i.e., bifidogenic activity) that were maintained even after pre-digestion in acidic conditions.

## 1. Introduction

It is well documented that the acquisition and shaping of infant microbiota strictly influence the well-being of newborns and pose the basis for long-term health [1,2,3].

Many studies correlate the composition of the human microbiota, especially the gut microbiota, to local and systemic diseases, suggesting that its correct development is necessary for optimal immune system maturation [4,5]. The establishment of the neonatal microbiota is affected by both environmental and host factors [1]. Indeed, at birth, infants come into contact with various microorganisms, especially those of maternal origin, that can stably and durably colonize the newborns mucosae, and it has been reported that maternal bacteria can be retrieved in infants’ feces [2]. Newborns’ health is also influenced by the birth delivery route since it affects vertical transmission. Newborns delivered vaginally show high levels of *Lactobacillus* in the mouth, skin, and gut [3]; in particular, the composition of gut microbiota resembles the vaginal one and is dominated by lactobacilli followed by *Sneathia* and *Prevotella* spp. [2]. On the other hand, children born via cesarean-section (C-section) show low microbial diversity in the gut and *Staphylococcus* dominance, which have been related to increased risk of developing various disorders, including asthma, juvenile arthritis, and inflammatory bowel disease [6,7].

In addition, infant gut microbiota development is influenced by the feeding mode. Maternal breast-feeding contributes to infant health providing nutrients as well as bioactive molecules and antibacterial agents, favoring the correct colonization of the intestine. Human milk oligosaccharides (HMOs) delivered by breast milk can selectively shape the infant gut microbiota, favoring the colonization of *Bifidobacterium* spp., while the gut microbiota of formula-fed babies also include *Bacteroides* [8].

With this in mind, the supplementation with beneficial microbes of vaginal origin in infants born via C-section or fed with formula could represent a valid strategy to drive the correct gut microbiota shaping. 

In the landscape of probiotics and functional strains, the assessment of their safety profiles is becoming a fundamental and urgent issue, along with demonstrating the ability to confer health benefits to the host. Indeed, the current widespread oral consumption of live probiotics is not completely risk-free because cases of systemic infections due to translocation have been reported in vulnerable patients and pediatric populations and the possibility of antibiotic resistance genes acquisition has not been totally excluded [9,10,11]. Even for well-known probiotics, such as strains belonging to *Lactobacillus* and related genera, a thorough analysis and understanding of potential risks must be undertaken, especially regarding antibiotic resistance [12].

International authorities and agencies have proposed safety assessment approaches for non-drug probiotic products [9]. As an example, in the European Union, the European Food Safety Authority (EFSA) periodically reviews the list of microorganisms considered as QPS (Qualified Presumption of Safety) and defines the guidelines for strains to be included in food products [13]. The risk assessment process required for pre-market authorization of novel microbial strains comprises the search for virulence factors and antibiotic resistance genes in the genome [13].

Another frontier in the probiotic field consists in the replacement of live beneficial cells with cell constituents, fractions or functional bioactive compounds that are still able to confer health-promoting effects to the host. Such cellular derivatives are generally referred to as postbiotics, although a unique consensus definition has not been defined yet [14,15]. Among these, inactivated or killed cells of beneficial strains are gaining much attention, since their employment offers the possibility to maintain functionality while improving safety profiles and reducing potential risks, especially those related to the transfer of genetic elements bearing antibiotic resistance [16]. The aim of the present paper was to analyse the genomic features of the vaginal strain *Limosilactobacillus vaginalis* BC17 and to characterize the bifidogenic and antipathogenic effect of heat-killed cells. The latter were also included in sunflower oil as a proof of concept of an oral formulation able to retain the beneficial properties of heat-killed cells.

## 2. Materials and Methods

### 2.1. Microorganisms and Culture Conditions

*L. vaginalis* BC17 was isolated from a vaginal swab collected from a healthy pre-menopause Caucasian woman, according to the protocol approved by the Ethics Committee of the University of Bologna, Bologna, Italy (52/2014/U/Tess) [17]. *Bifidobacterium* strains (*B. breve* DSM20091, *B. breve* DSM20456, *B. bifidum* DSM20082, *B. bifidum* DSM20213, *B. bifidum* DSM20215, *B. longum* subsp. *longum* DSM20219, *B. longum* subsp. *infantis* DSM20088, *B. longum* subsp. *infantis* DSM20090, *B. adolescentis* DSM20083, *B. adolescentis* DSM20086, *B. angulatum* DSM20098) were purchased from DSMZ (Braunschweig, Germany) [18]. *L. vaginalis* and bifidobacteria were routinely cultured in de Man, Rogosa, and Sharpe broth (MRS, Difco, Detroit, MI, USA) supplemented with L-cysteine 0.05% (*w*/*v*) (Merck, Milan, Italy) at 37 °C, in anaerobic jars containing Gas-Pak EZ (Beckton, Dickinson and Co., Milan, Italy).

*Escherichia coli* SO107 and *Streptococcus agalactiae* SO104 were isolated at Sant’Orsola-Malpighi University Hospital of Bologna (Italy) during routine diagnostic procedures [18,19]. Enterotoxigenic *E. coli* (ETEC), *Salmonella enterica*, *Yersinia enterocolitica,* and *Enterococcus faecium* BC105 belong to the Department of Pharmacy and Biotechnology of University of Bologna [19,20]. These strains were aerobically grown at 37 °C in Brain Heart Infusion (BHI, Difco).

### 2.2. L. vaginalis BC17 Whole Genome Sequencing and Annotation

*L. vaginalis* BC17 was grown for 24 h, after which 2 mL of culture was centrifuged for 10 min at 10,000× *g* (Microfuge 16, Beckman Coulter, Milan, Italy), and genomic DNA was extracted using the DNeasy Blood and Tissue Kit (Qiagen, Hilden, Germany). DNA was quantified via the Qubit dsDNA BR assay kit using the Qubit 4.0 fluorometer (Life Technologies, Monza, Italy) and sequenced by Oxford Nanopore Whole Genome Sequencing technology: the genomic library was prepared using the SQK-LSK109 and EXP-NBD104 kits (Oxford Nanopore Technologies, Oxford, UK), loaded on a R9.4.1 flowcell (MIN106D, Oxford Nanopore Technologies, Oxford, UK) and sequenced with the GridION Nanopore device (Oxford Nanopore Technology, Oxford, UK). The genome was de novo-assembled using Canu assembler v2.2 [21] and annotated by Bakta v1.4.1 [22] with a genome coverage of 1231.05X.

### 2.3. L. vaginalis BC17 Genome Analysis: Search for Virulence Factors and Antibiotic Resistance Genes

The overall quality of *L. vaginalis* BC17 genomic sequence was checked using the genomic platform CLC Genomics Workbench 22 (Qiagen), and species identification was performed through in silico MLST using the online tool autoMLST (http://automlst.ziemertlab.com/, accessed on 30 January 2023). The presence of genetic elements potentially leading to antibiotic resistance was investigated by using ResFinderFG 2.0 (https://cge.food.dtu.dk/services/ResFinderFG/, accessed on 30 January 2023) with the stringency set to 80% ID and minimum length to 60%. To evaluate the presence of genetic elements leading to virulence factors, *L. vaginalis* BC17 ORFs were investigated against the VFDB database (http://www.mgc.ac.cn/VFs/, accessed on 30 January 2023) through a multiblast approach (parameters: Word size: 3; Max E-value: 0,1; Max no of hits: 1).

### 2.4. L. vaginalis BC17 Antimicrobial Susceptibility Testing

Antimicrobial susceptibility testing (AST) was performed by a broth microdilution assay adapted from [23]. Starting from powder stocks, all the drugs (i.e., Ampicillin, Clindamycin, Tetracycline, Vancomycin, Gentamycin, Erythromycin, Kanamycin, and Streptomycin) were initially resuspended in distilled water or DMSO following manufacturer’s instructions. *L. vaginalis* BC17 suspension was prepared in Wilkins–Chalgren Anaerobe broth (ThermoFisher, Monza, Italy) at a final concentration of 3 × 10^6^ CFU/mL. Each well of a 96-well flat-bottom plate (Corning Inc., Pisa, Italy) was inoculated with 50 μL of *L. vaginalis* BC17 suspension and with 50 μL of the antimicrobial agent, serially two-fold diluted in Wilkins–Chalgren Anaerobe broth (256–0.03 μg/mL). Afterwards, the microdilution tray was incubated in anaerobic conditions for 48 h at 37 °C. The minimum inhibitory concentration (MIC) was considered as the lowest drug concentration giving a complete inhibition of bacterial growth. Antimicrobial susceptibility testing was carried out in duplicate.

### 2.5. Heat Inactivation of L. vaginalis BC17 Cells

An overnight culture of *L. vaginalis* BC17 (10 mL) was transferred in 100 mL of MRS broth and allowed to grow for additional 24 h. Then, the cell pellet was harvested by centrifugation (10,000× *g* for 10 min, Centrisart G-16C, Sartorius, Goettingen, Germany), washed, and resuspended in 5 mL of sterile saline (NaCl 0.9%, *w*/*v*). The inactivation was achieved by immersing *L. vaginalis* BC17 suspension into hot water bath (Falc, Bergamo, Italy) at 70 °C for 2 h and then cooling it at 4 °C for 24 h; heating and cooling steps were repeated twice on each sample. *L. vaginalis* BC17 suspension was seeded on MRS plates, before and after each heat-treatment, to determine the initial number of cells and monitor cell viability. Three batches of heat-killed *L. vaginalis* BC17 cells (HK-BC17) were obtained from three independent cultures.

### 2.6. Bifidogenic and Antimicrobial Activity of Heat-Killed L. vaginalis BC17 Cells 

HK-BC17 was sought for its ability to stimulate the proliferation of 11 *Bifidobacterium* strains (listed above). The effects of HK-BC17 on the planktonic growth of pathogens (i.e., *E. coli* SO107, ETEC, *S. enterica*, *Y. enterocolitica, E. faecium* BC105, and *S. agalactiae* SO104) were also evaluated. Three batches of HK-BC17 were analyzed in triplicate on three independent assays.

Microorganisms were subcultured twice as previously described and subsequently diluted in 2X appropriate medium (MRS or BHI) to achieve a final concentration of 10^6^ CFU/mL. HK-BC17 was then diluted in sterile saline to obtain suspensions at three concentrations (corresponding to 2 × 10^7^, 2 × 10^6^ and 2 × 10^5^ CFU/mL). Suspensions of bifidobacteria (100 μL) were used to inoculate 96 multi-well round-bottom plates (Corning Inc.), while suspensions of pathogens (100 μL) were inoculated in 96 multi-well flat-bottom plates (Corning Inc.). To these wells, 100 μL of HK-BC17 suspensions was added to test final concentrations corresponding to 10^7^, 10^6^ and 10^5^ CFU/mL.

Microbial suspension (100 μL) inoculated with 100 μL of sterile saline served as the positive control. Wells containing growth medium (2X MRS or BHI) together with sterile saline or HK-BC17 at different concentrations were used as blanks and sterility controls. 

After 24 h of incubation at 37 °C under anaerobic conditions, the growth of bifidobacteria and pathogens was established by reading the absorbance at 600 nm (EnSpire Multimode Plate Reader, PerkinElmer Inc., Waltham, MA, USA). The planktonic growth of bifidobacteria and pathogens in the presence of HK-BC17 was calculated as percentages relative to the absorbance of the corresponding positive controls (considered as 100%).

### 2.7. Effects of Heat-Killed L. vaginalis BC17 Cells on Biofilms of Bifidobacterium spp. and Pathogens

The impact of HK-BC17 (at the highest concentration, corresponding to 10^7^ CFU/mL) on microbial biofilms was evaluated considering two mechanisms of action, namely (i) the effects on the formation of biofilm and (ii) the effects on pre-formed biofilms of *Bifidobacterium* spp. and pathogens. Three batches of HK-BC17 were analyzed in triplicate on three independent assays. 

Bifidobacteria and pathogens were diluted in cultural broth (2X MRS or BHI) at 10^7^ CFU/mL and used as inoculum for all biofilm assays. To assess the effects on the formation of biofilms, microorganisms were inoculated (100 μL) in 96 multi-well plates (Corning Inc., Pisa, Italy) with 100 μL of HK-BC17 at 2 × 10^7^ CFU/mL. The biofilms were allowed to form for 48 h under anaerobic (*Bifidobacterium* spp.) or aerobic (pathogens) conditions.

To evaluate the impact on pre-formed biofilms, microorganisms were first inoculated (200 μL) in the wells, and biofilms were developed for 48 h. Afterwards, supernatants were removed, adherent cells were washed once with sterile saline, and wells were filled with 100 μL of HK-BC17 (2 × 10^7^ CFU/mL) and 100 μL of 2X medium. Plates were then incubated for an additional 24 h.

Biofilms formed in the absence of HK-BC17 were the positive controls. Blanks containing 2X MRS/BHI broths and sterile saline or HK-BC17 were also included.

For each experiment, biofilms were detected by crystal violet staining and MTT assay, which enable the quantification of total biofilm biomass and biomass metabolic activity, respectively [19]. Crystal violet staining was performed following the previously reported protocol [18]. Briefly, culture supernatants were removed, adherent cells were washed twice with sterile saline, fixed with 200 μL of absolute ethanol (Merck), and stained with crystal violet (CV, Merck) for 10 min. After washing wells three times, the dye bound to adherent cells was resolubilized in 200 μL of ethanol and the absorbance was measured at 595 nm by a microplate reader (EnSpire Multimode Plate Reader).

The MTT assay was carried out as reported by Giordani et al. [19]. After removing the supernatants, the adherent biofilms were washed twice and incubated in the dark with 100 μL of 3-(4,5-dimethyl-2-thiazolyl)-2,5-diphenyl-2H-tetrazolium bromide (MTT, Merck) (0.5 mg/mL in culture medium) at 37 °C for 4 h. Afterwards, the medium was removed from the wells and replaced with 200 μL of isopropanol (Merck) to solubilize the formazan crystals formed, which were quantified by reading the absorbance at 570 nm (EnSpire Multimode Plate Reader).

The biofilm formation of bifidobacteria and pathogens in the presence of HK-BC17 was calculate as percentages relative to the absorbances of the corresponding positive controls (considered as 100%).

### 2.8. Preparation of L. vaginalis BC17 Oil Suspension 

*L. vaginalis* BC17 cells, inactivated as previously reported, were freeze-dried at 0.1 mbar and −47 °C (Christ Freeze Dryer ALPHA 1–2, Milan, Italy). The lyophilized HK-BC17 powder (L-HK-BC17, corresponding to 10^12^ CFU/g) was accurately weighed using an analytical balance and added to sunflower oil (ACEF, Piacenza, Italy) at a final concentration of 1% (*w/w*). A complete dispersion of L-HK-BC17 in oil was achieved by gentle mixing at 300 rpm for 150 min at room temperature. Three batches were produced and stored in dark bottles at +4–8 °C. 

### 2.9. Technological Characterization of L. vaginalis BC17 Oil Suspension

L-HK-BC17 oil suspension was observed at fixed times (14, 30 and 90 days) to monitor possible physical changes, such as modifications in color and odor. Color modification was evaluated by visually inspecting the suspension withdrawn from the storage conditions. The odor was monitored by olfactory examination for the possible changes or the presence of unpleasant smell. Furthermore, for each batch, an aliquot of L-HK-BC17 oil suspension was placed inside a 10 mL graduated cylinder and allowed to stand for 24 h. The sedimentation volume was determined by measuring the volume of the sediment occupied by L-HK-BC17 powder under the influence of gravity and it was recorded at intervals of 1, 14, 30, and 90 days. The sedimentation volume (F) was calculated using the following formula:

F = Vu/V0

where Vu is the final volume of sediment and V0 is the original volume of suspension [24]. Furthermore, L-HK-BC17 oil suspension was evaluated for re-dispersibility after 1, 14, 30, and 90 days by turning the bottle through a 180-degree cycle. Re-dispersibility was recorded as the number of inversions required to completely resuspend the sediment [25]. The viscosity of the formulated suspension was measured after sediment redispersion using the rotational viscometer (spindle TR8, 200 rpm; Visco Star, Fungilab S.A., Barcelona, Spain) at different time intervals (0, 1, 14, 30, and 90 days). All measurements were carried out on the three batches.

### 2.10. Functional Characterization of L. vaginalis BC17 Oil Suspension

The activities of L-HK-BC17 and freshly prepared L-HK-BC17 oil suspension were assessed toward planktonic growth of *Bifidobacterium* spp. and their biofilms (by CV staining), following protocols described in the previous sections. L-HK-BC17 powder and L-HK-BC17 oil suspension were also checked for their bifidogenic activity against free-floating bifidobacteria after 30 and 90 days of storage at +4–8 °C.

For testing, L-HK-BC17 was resuspended in sterile saline, while 1 g of L-HK-BC17 oil suspension was first mixed with 0.5 g of Tween 80 (Merck) and then added to 9.5 mL of sterile saline and thoroughly vortexed for 10 min to form an oil-in-water emulsion. Both L-HK-BC17 and emulsion were further diluted in sterile saline to test the same concentrations as for HK-BC17. Sunflower oil was treated in the same way and tested as comparison.

To mimic the passage through the acidic stomach, 1 g of L-HK-BC17 oil suspension with 2 mL of simulated gastric fluid (SGF) (125 mM NaCl, 7 mM KCl, 45 mM NaHCO_3_, 3 g/L pepsin, pH = 3) was incubated under agitation (100 rpm, Certomat^®^Sartorius AG, Göttingen, Germany) for 2 h at 37 °C [18]. Afterwards, the oil-in-water emulsion was formed by adding 0.5 g of Tween 80 and 7.5 mL of sterile saline, the pH was adjusted to 6.5 (with NaOH 5 M), and the effects on the planktonic cultures and biofilms of bifidobacteria and pathogens were assessed.

### 2.11. Statistical Analysis

Results were expressed as mean ± standard deviation (SD). Student *t* test was used for the comparison of two means, and one-way ANOVA followed by Tukey’s correction was used for multiple comparison. All statistical analyses were performed using GraphPad Prims version 9.5.1 for Windows (GraphPad Software, San Diego, CA, USA, www.graphpad.com). Differences were deemed significant for *p* < 0.05.

## 3. Results

### 3.1. L. vaginalis BC17 Genome Analysis

The whole genome sequence of *Limosilactobacillus vaginalis* BC17 strain (GenBank accession number CP122957) was obtained by using Oxford Nanopore Technology, resulting in a genome coverage of 1231.05X. This sequence reveals an average nucleotide identity (ANI%) of 99.1% when compared to *Limosilactobacillus vaginalis* type strain DSM 5837, thereby confirming species identification previously reported for BC17 isolate [17]. The genomic sequence is 1870 536 bp long and has a G+C content of 40.6%, in line with reference genomes; the analysis did not highlight plasmidic DNA. Genome annotation analysis identified a total of 1943 coding sequences and 109 RNA genes, including 62 tRNAs, 15 rRNAs, and 31 ncRNAs. According to the Bakta Annotation pipeline, 45 proteins were identified as domains of unknown function and 426 were identified as hypothetical proteins. 

In order to assess strain’s safety, *L. vaginalis* BC17 genome was searched for sequences conferring antibiotic resistance and virulence factors, following EFSA guidelines for microorganisms intentionally used in the food chain [13]. Special attention was given to genes related to antibiotic resistance and virulence factors found in lactobacilli, reviewed by Colautti et al. [12], which were also manually searched in the annotated genome. The absence of positive hits suggests that, from a genetic perspective, *L. vaginalis* BC17 can be considered safe. In addition, genes possibly related to exopolysaccharides (EPS) synthesis were highlighted: *L. vaginalis* BC17 genome possess a bacterial sugar transferase, three glycosiltranferases, a polysachharide biosynthesis protein, and the UDP-galactopyranose mutase (*glf*). All these genes mapped in the region 1,198,670–1,214,186, suggesting the presence of an EPS gene cluster.

### 3.2. L. vaginalis BC17 Antimicrobial Susceptibility Testing

The absence of acquired genes related to antibiotic resistance, as highlighted by the analysis of *L. vaginalis* BC17 genome, was further confirmed at the phenotypic level through antimicrobial susceptibility testing on the following antibiotics, i.e., Ampicillin, Clindamycin, Tetracycline, Vancomycin, Gentamicin, Erythromycin, Kanamycin, Streptomycin, and minimum inhibitory concentrations (MICs) were assessed. The MIC values (expressed as μg/mL) are reported in Table 1. As for other lactobacilli, *L. vaginalis* BC17 possessed an intrinsic resistance to vancomycin (MIC > 256 μg/mL). For all the other antimicrobials, the MIC values were found to be below the microbiological cut-off values set by the EFSA for *Limosilactobacillus reuteri* and other lactobacilli [26], suggesting the substantial safety (i.e., absence of acquired resistance mechanisms) of *L. vaginalis* BC17 strain.

### 3.3. Heat Inactivation of L. vaginalis BC17 Cells and Lyophilization

*L. vaginalis* BC17 cells were suspended in 5 mL of sterile saline at a concentration of 8 × 10^10^ viable cells/mL (equivalent to 4 × 10^11^ viable cells, before heat treatment). Two cycles of heating followed by overnight storage at 4 °C were required for heat inactivation. Specifically, after the first heat treatment viability decreased to 1–2 × 10^4^ viable cells/mL, while after the second heat treatment a complete abolishment of *L. vaginalis* BC17 viability was achieved. The freeze-drying of the resulting heat-killed *L. vaginalis* BC17 (HK-BC17) suspension made it possible to obtain 0.4 g of lyophilized powder. Thus, the lyophilized product (L-HK-BC17) was characterized by high HK-BC17 cell density, equivalent to 10^12^ heat-killed cells/g. 

### 3.4. Heat-Inactivated L. vaginalis BC17 Cells Promote Bifidobacterium spp. Growth

**Figure 1:**
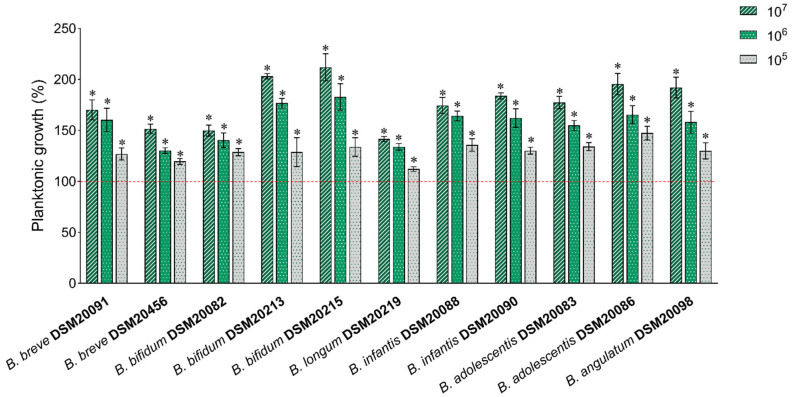
Bifidogenic activity of HK-BC17. The effects of HK-BC17 at three concentrations (10^7^, 10^6^ and 10^5^ heat-killed cells/mL) on planktonic cultures of *Bifidobacterium* spp. are expressed as percentages with respect to the control (100%, red line) (mean ± SD, *n* = 3); *, *p* < 0.05.

HK-BC17 was first sought for its ability to stimulate the planktonic growth of 11 *Bifidobacterium* strains that belong to species highly represented in the human colon, i.e., *B. breve* DSM20091, *B. breve* DSM20456, *B. bifidum* DSM20082, *B. bifidum* DSM20213, *B. bifidum* DSM20215, *B. longum* subsp. *longum* DSM20219, *B. longum* subsp. *infantis* DSM20088, *B. longum* subsp. *infantis* DSM20090, *B. adolescentis* DSM20083, *B. adolescentis* DSM20086, *B. angulatum* DSM20098 [27]. Three batches of HK-BC17 were analyzed in triplicate in three independent assays. Three concentrations of HK-BC17 were investigated (10^7^, 10^6^, and 10^5^ heat-killed cells/mL) and the results are depicted in Figure 1. HK-BC17 at a concentration of 10^7^ heat-killed cells/mL effectively stimulated the proliferation of all tested bifidobacteria (*p* < 0.05), with differences depending on the *Bifidobacterium* strain. In particular, *B. bifidum* DSM20213 and *B. bifidum* DSM20215 were highly influenced by HK-BC17 at 10^7^ heat-killed cells/mL, showing growth percentages exceeding 200% compared to the control (203% and 212%, respectively). *B. longum* subsp. *longum* DSM20219, *B. bifidum* DSM20082 and *B. breve* DSM20456 were the less stimulated strains (growth of 142–151%). The growth of other bifidobacteria strains incubated with HK-BC17 varied between 170% (*B. breve* DSM20091) and 195% (*B. adolescentis* DSM20086).

The stimulating effect was clearly dose-dependent, as it significantly decreased with the reduction in the HK-BC17 concentration (*p* < 0.05). Nevertheless, a pronounced bifidogenic activity was observed even in the presence of HK-BC17 at 10^6^ heat-killed cells/mL (*p* < 0.05), with *Bifidobacterium* spp. growth ranging between 130% (*B. breve* DSM20456) and 183% (*B. bifidum* DSM20215). HK-BC17 at 10^5^ heat-killed cells/mL was still able to significantly promote the proliferation of all bifidobacteria (growth percentages of 112–147%), although to a lesser extent, indicating that a small number of inactivated cells was sufficient to elicit a beneficial response.

### 3.5. Heat-Inactivated L. vaginalis BC17 Cells Promote Bifidobacterium spp. Biofilms

To further investigate the bifidogenic potential of HK-BC17, the concentration of 10^7^ heat-killed cells/mL was selected for biofilm assays. Since microorganisms are mainly present as biofilms under physiological conditions rather than as free-floating cells [28], the ability to promote bifidobacteria growth even in the biofilm form represents an added value for a postbiotic preparation. 

**Figure 2:**
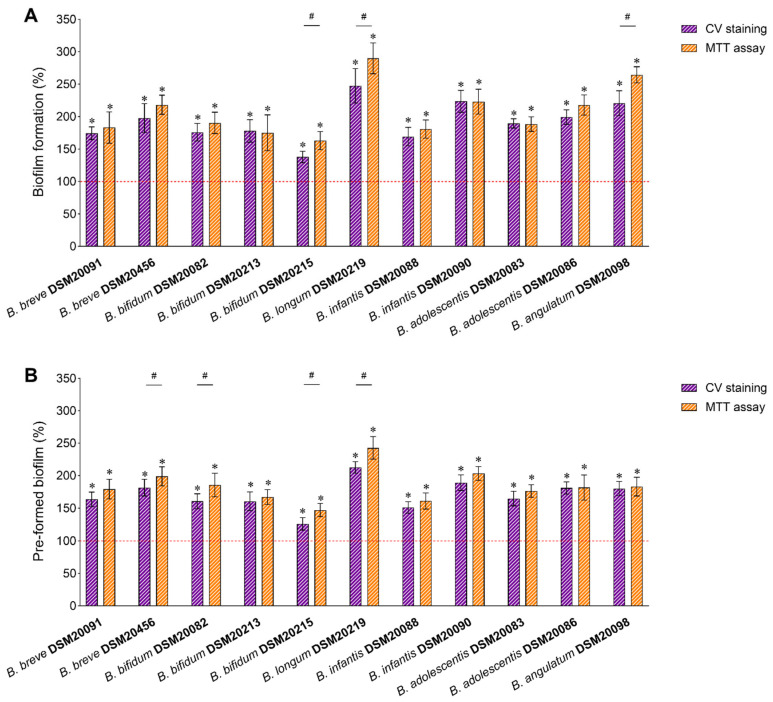
Effects of HK-BC17 (10^7^ heat-killed cells/mL) on biofilms formation (**A**) and pre-formed biofilms (**B**) of *Bifidobacterium* spp. Biofilms are quantified by CV staining and MTT assay. Results are expressed as percentages with respect to the control (100%, red line) (mean ± SD, *n* = 3); *, *p* < 0.05. Statistical differences between the two methods are also shown; #, *p* < 0.05.

The biofilm-stimulating capability of HK-BC17 was evaluated by applying two complementary methods. Specifically, the total biofilm biomass, comprising extracellular matrix along with live and dead cells, was quantified by crystal violet (CV) staining, while the MTT assay was used to detect only viable and metabolically active cells. The results for biofilm formation of *Bifidobacterium* spp. and pre-formed biofilms are reported in Figure 2A and Figure 2B, respectively. 

The data clearly indicated that the presence of HK-BC17 effectively increased the formation of biofilm biomass of all bifidobacteria considered (*p* < 0.05) (Figure 2A, CV staining), with an average biofilm formation of 192% compared to the control. Additionally, the viability of bifidobacteria was enhanced when biofilms were formed in the presence of HK-BC17 (*p* < 0.05) (Figure 2A, MTT assay), with an average biofilm formation of 209%. 

The susceptibility of bifidobacteria to the action of HK-BC17 greatly varied among different strains and was not species-specific. In particular, five *Bifidobacterium* strains were particularly responsive to HK-BC17, reaching bifidobacteria biofilm biomass of 198–247% and bifidobacteria viability in biofilms of 218–290%. These belong to the species *B. longum* subsp. *longum* (DSM20219), *B. longum* subsp. *infantis* (DSM20090), *B. angulatum* (DSM20098), *B. adolescentis* (DSM20086), and *B. breve* (DSM20456).

The formation of *B. bifidum* DSM20215 biofilm was slightly stimulated by HK-BC17 (138%/163% by CV staining/MTT assay). However, other *B. bifidum* strains (DSM20082 and DSM20213) incubated with HK-BC17 showed biofilm formation percentages as high as 178% with CV staining and 190% with MTT assay.

Notably, even the pre-formed biofilms of all *Bifidobacterium* spp. were considerably improved after treatment with HK-BC17 (*p* < 0.05). The average biofilm formation percentages were 170% and 184% considering the promotion of biomass formation (Figure 2B, CV staining) and bifidobacteria viability in biofilms (Figure 2B, MTT assay), respectively. Consistent with what was previously observed, the biofilms of *B. longum* subsp. *longum* DSM20219, *B. longum* subsp. *infantis* DSM20090, *B. breve* DSM20456, *B. adolescentis* DSM20086, and *B. angulatum* DSM20098 were the most influenced by HK-BC17, reaching percentages of 180–213% by CV staining and 182–243% by MTT assay. Biofilms of *B. breve* DSM20215 were confirmed to be the least affected by HK-BC17 (126%/147% by CV staining/MTT assay), while treatment of the other pre-formed bifidobacteria biofilms allowed percentages of 151–165% (CV staining) and 161–179% (MTT assay).

Considering the overall results collected by both CV staining and MTT assay, HK-BC17 was more effective in stimulating the formation of bifidobacteria biofilms than the biofilms already established (*p* < 0.05).

Furthermore, to assess the agreement between the two methods, a correlation analysis between datasets collected by CV staining and MTT assay was conducted. The Person’s correlation coefficient was found to be 0.9361, indicating an excellent correlation between the two methods employed for biofilm quantification. The percentages detected by the MTT assay were significantly higher than those obtained by the CV assay (*p* < 0.05) for only 3 bifidobacteria out of 11 in the biofilm formation assay (Figure 2A) and for 4 bifidobacteria out of 11 in the pre-formed biofilms assay (Figure 2B).

It is worth noting that *B. bifidum* DSM20215, whose planktonic culture was the most stimulated by HK-BC17, was instead poorly enhanced when grown in biofilm form. On the contrary, HK-BC17 greatly promoted biofilm of *B. longum* DMS20219 but had a lesser effect on its planktonic form. These results suggested that HK-BC17 prebiotic activity depends on the specific *Bifidobacterium* strain tested, rather than its mode of growth (planktonic/biofilm).

### 3.6. Effects of Heat-Inactivated L. vaginalis BC17 on Human Pathogens

To exclude undesired stimulating effects and to investigate a potential antimicrobial activity, HK-BC17 was also tested toward opportunistic (i.e., *Escherichia coli* SO107, *Enterococcus faecium* BC105 and *Streptococcus agalactiae* SO104) and virulent (i.e., Enterotoxigenic *E. coli*, *Salmonella enterica* and *Yersinia enterocolitica*) pathogens. 

**Figure 3:**
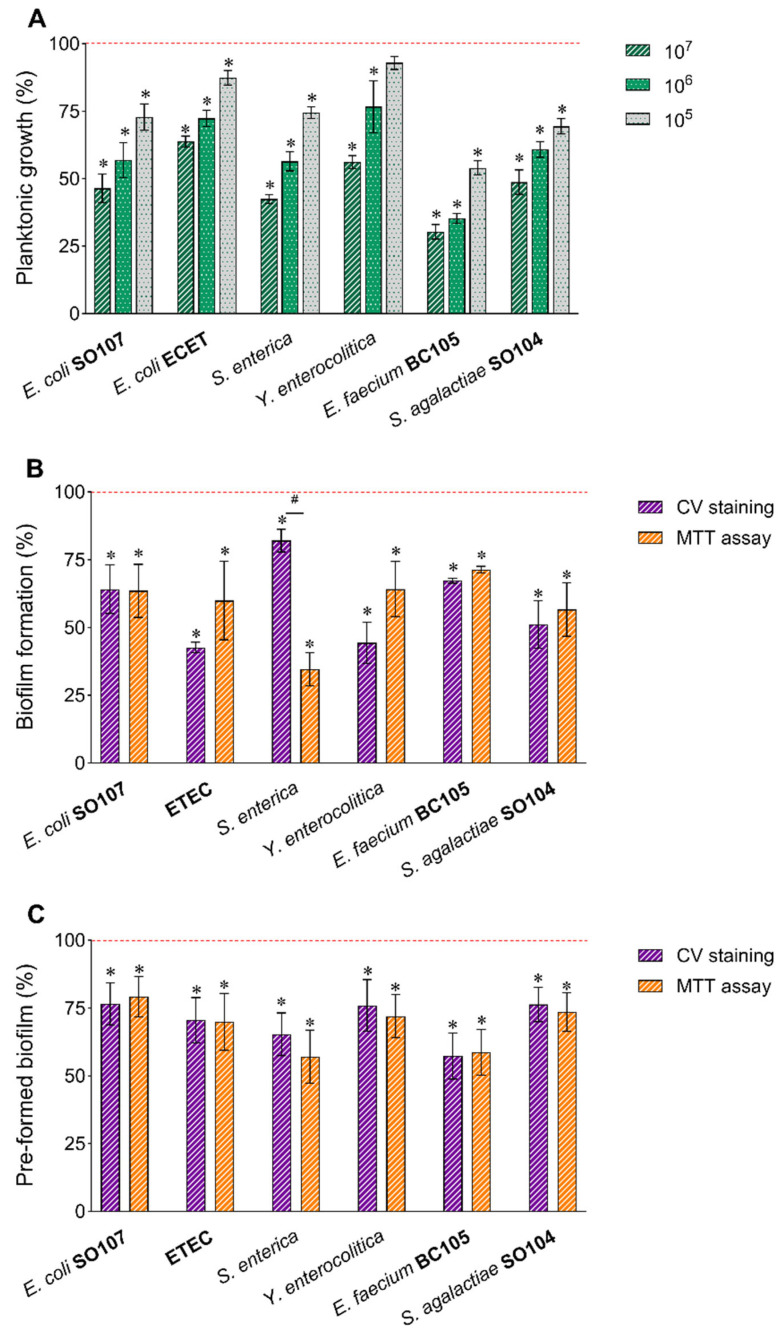
Effects of HK-BC17 on human pathogens. Effects of HK-BC17 at three concentrations (10^7^, 10^6^ and 10^5^ heat-killed cells/mL) on planktonic cultures of *E. coli* SO107, enterotoxigenic *E. coli* (ETEC), *S. enterica, Y. enterocolitica, E. faecium* BC105 and *S. agalactiae* SO104 (**A**). Effects of HK-BC17 (10^7^ heat-killed cells/mL) on biofilms formation (**B**) and pre-formed biofilms (**C**) of the same pathogens quantified by CV staining and MTT assay. Results are expressed as percentages with respect to the control (100%, red line, mean ± SD, *n* = 3); *, *p* < 0.05. Statistical differences between CV and MTT methods are also shown; #, *p* < 0.05.

Importantly, HK-BC17 did not exert any stimulating activity on these strains, neither for what concerns planktonic growth nor for biofilm formation. On the contrary, HK-BC17 significantly reduced pathogens’ growth between 36% (ETEC) and 70% (*E. faecium* BC105) at a concentration of 10^7^ heat-killed cells/mL (Figure 3A). Although the effect was dose-dependent, the antimicrobial activity was still evident at 10^6^ heat-killed cells/mL (inhibition rates of 28–65%) and at 10^5^ heat-killed cells/mL, for which five out of six pathogens significantly inhibited (inhibition rates of 13–46%).

Moreover, HK-BC17 at 10^7^ heat-killed cells/mL exerted a mild anti-biofilm activity, being able to inhibit pathogens’ biofilm formation (biofilm inhibition of 18–57%/29–65%, quantified by CV staining/MTT assay, Figure 3B) and to disrupt pre-formed biofilms (biofilm eradication of 23–43%/21–43% quantified by CV staining/MTT assay, Figure 3C).

### 3.7. Preparation and Technological Characterization of L. vaginalis BC17 Oil Suspension 

The L-HK-BC17 oil suspension was prepared to bear 10^9^ heat-killed cells of HK-BC17 per dose (estimated to be about 0.1 g of suspension, equivalent to five drops). To monitor the occurrence of undesirable physical changes, the prepared L-HK-BC17 oil suspension was checked over a period of 90 days. The oral suspension exhibited a faint yellow color and a characteristic odor reminiscent of sunflower seeds. Visual inspection revealed that no color modification occurred during the selected storage period. Additionally, no changes in odor were noticed, demonstrating the good physical stability of the dosage form. Moreover, the formulated suspension showed an average sedimentation volume of 0.13 ± 0.01 after 24 h, which was maintained over the storage period (*p* > 0.05), and it presented suitable redispersion ability. In fact, the number of inversions required to completely resuspend the formulation was 30 ± 6, 32 ± 5, 39 ± 5 and 40 ± 4 after 1, 14, 30, and 90 days, respectively. Finally, the viscosity of the prepared formulations was equal to 60 mPa*s, and no significant variation in viscosity was observed during the storage period.

### 3.8. Functional Characterization of L. vaginalis BC17 Oil Suspension 

**Figure 4:**
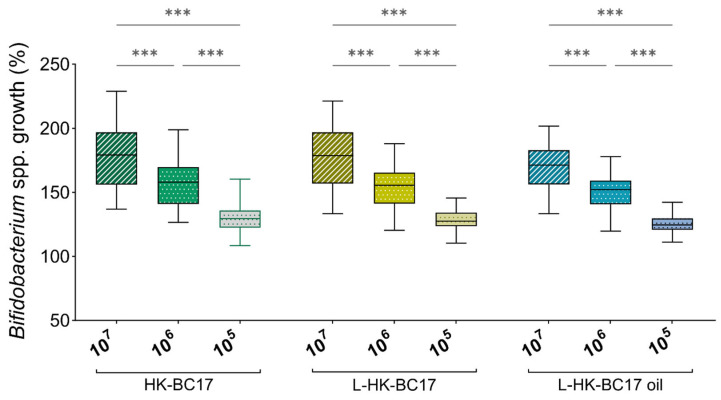
Effects of HK-BC17, L-HK-BC17 and L-HK-BC17 oil on planktonic cultures of *Bifidobacterium* spp. HK-BC17, L-HK-BC17 and L-HK-BC17 oil were tested at three concentrations (10^7^, 10^6^ and 10^5^ heat-killed cells/mL). Each box represents the interquartile range (25th and 75th percentiles), lines within the boxes indicate the median values for the samples. The extremes of the bars indicate the minimum and maximum values, respectively.***, *p* < 0.001.

**Figure 5:**
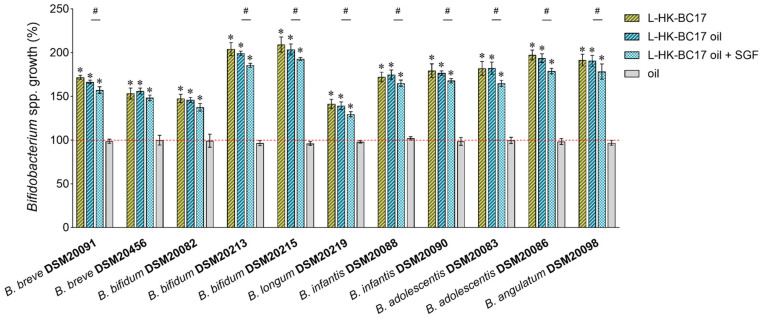
Bifidogenic activity of formulated HK-BC17. The effects of sunflower oil, L-HK-BC17, L-HK-BC17 oil (the not-digested and digested in simulated gastric fluid (SGF)) (10^7^ heat-killed cells/mL) on planktonic cultures of *Bifidobacterium* spp. are expressed as percentages with respect to the control (100%, red line) (mean ± SD, *n* = 3). *, *p* < 0.05. Statistical differences between the not-digested and digested L-HK-oil are also shown; #, *p* < 0.05.

The functional activity of the L-HK-BC17 and L-HK-BC17 oil suspension was investigated in terms of their ability to stimulate *Bifidobacterium* spp. and inhibit Gram-positive and Gram-negative pathogens cultured as free-floating cells and biofilms. Data regarding the stimulation of *Bifidobacterium* planktonic cultures at three concentrations (10^7^, 10^6^ and 10^5^ heat-killed cells/mL) are depicted in the boxplot in Figure 4, which shows the overall results obtained for the L-HK-BC17 and L-HK-BC17 oil suspension toward all bifidobacteria. The results obtained with the not-formulated HK-BC17 were also included for comparison. The capability of the L-HK-BC17 and L-HK-BC17 oil suspension (10^7^ heat-killed cells/mL) to stimulate the growth of *Bifidobacterium* spp. is also extensively shown in Figure 5. As formerly observed for HK-BC17, the growth stimulating effect is dose-dependent (*p* < 0.05). Specifically, bifidobacteria showed growth percentages ranging from 142% (*B. longum* subsp. *longum* DSM20219) to 209% (*B. bifidum* DSM20215) when cultured in the presence of L-HK-BC17 (10^7^ heat-killed cells/mL) and from 139% (*B. longum* subsp. *longum* DSM20219) to 204% (*B. bifidum* DSM20215) when grown in the presence of the L-HK-BC17 oil suspension (10^7^ heat-killed cells/mL) (Figure 5). The median growth percentages decreased to 155/152% and to 125/128% for the L-HK-BC17/L-HK-BC17 oil suspension at 10^6^ and 10^5^ heat-killed cells/mL, respectively (Figure 4). Importantly, no significant differences in activity were found between the not-formulated HK-BC17, L-HK-BC17, and L-HK-BC17 oil suspension at all tested concentrations (Figure 4). This finding highlights that the technological processes, namely lyophilization and incorporation into sunflower oil, did not hinder the bifidogenic ability of HK-BC17.

**Figure 6:**
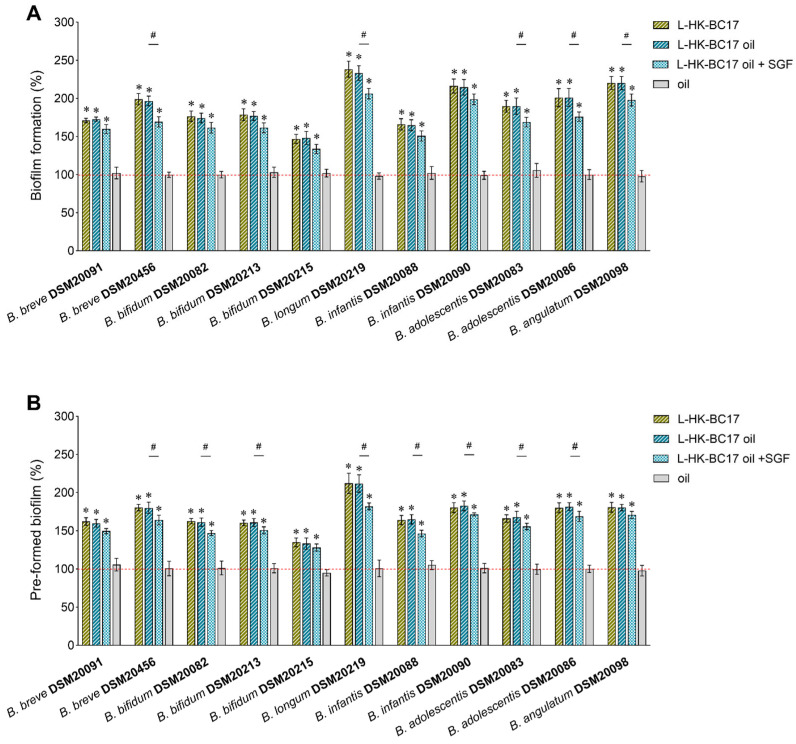
Biofilm-stimulating activity of formulated HK-BC17. The effects of sunflower oil, L-HK-BC17, L-HK-BC17 oil (the not-digested and digested in simulated gastric fluid (SGF)) (10^7^ heat-killed cells/mL) on biofilms formation (**A**) and pre-formed biofilms (**B**) of *Bifidobacterium* spp. are quantified by CV staining and expressed as percentages with respect to the control (100%, red line) (mean ± SD, *n* = 3). *, *p* < 0.05. Statistical differences between the not-digested and digested L-HK-BC17 oil are also shown; #, *p* < 0.05.

Furthermore, the L-HK-BC17 and L-HK-BC17 oil suspension (10^7^ heat-killed cells/mL) effectively stimulated the biofilms of all *Bifidobacterium* spp. tested. In particular, when biofilms were established in the presence of L-HK-BC17/L-HK-BC17 oil suspension, the formation percentages varied between 147/148% (*B. bifidum* DSM20215) and 238/233% (*B. longum* subsp. *longum* DSM20219) (Figure 6A). Biofilm formation percentages ranged from 133/135% (*B. bifidum* DSM20215) to 212/214% (*B. longum* subsp. *longum* DSM20219) when the L-HK-BC17 or L-HK-BC17 oil suspension were added to pre-formed biofilms (Figure 6B), consistent with the biofilm-stimulating effects exerted by the not-formulated HK-BC17. No significant differences were found between the L-HK-BC17 and L-HK-BC17 oil suspension (*p* > 0.05). Sunflower oil diluted in the same way as the L-HK-BC17 oil suspension did not affect the viability (Figure 5) and biofilms (Figure 6) of bifidobacteria compared to the control (*p* > 0.05), implying that this vehicle is compatible with these microbiota members.

**Figure 7:**
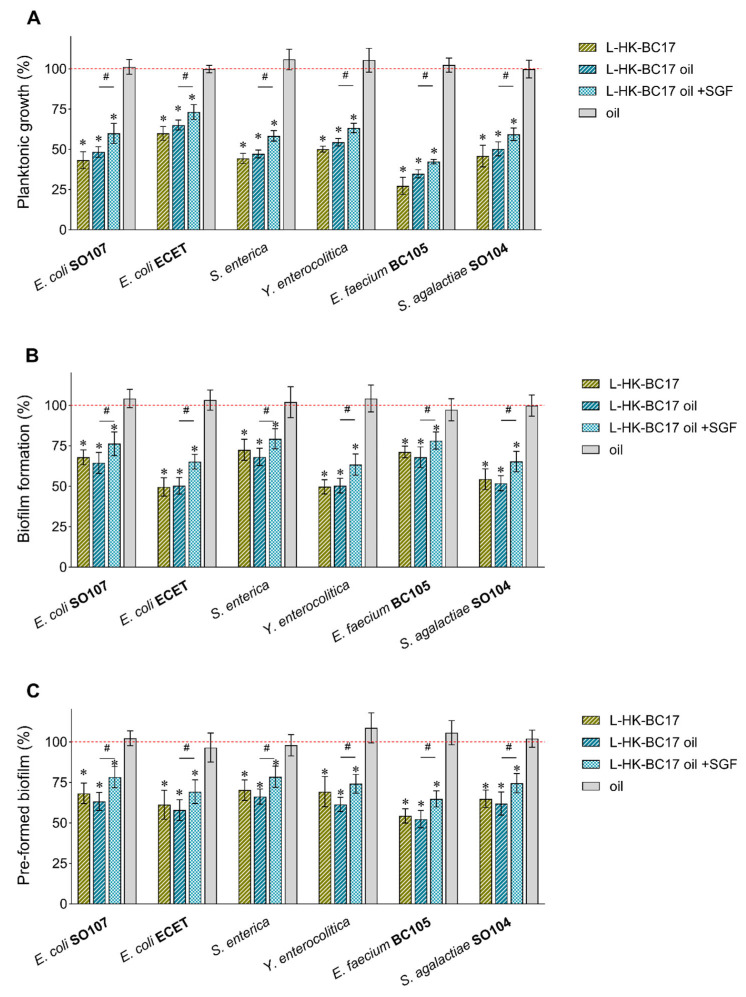
Effects of formulated HK-BC17 on human pathogens. Effects of sunflower oil, L-HK-BC17, L-HK-BC17 oil (the not-digested and digested in simulated gastric fluid (SGF)) (10^7^ heat-killed cells/mL) on planktonic cultures (**A**), biofilms formation (**B**), and pre-formed biofilms (**C**) of *E. coli* SO107, enterotoxigenic *E. coli* (ETEC), *S. enterica, Y. enterocolitica, E. faecium* BC105, and *S. agalactiae* SO104. Biofilms were quantified by CV staining. Results are expressed as percentages with respect to the control (100%, red line, mean ± SD, *n* = 3); *, *p* < 0.05 Statistical differences between the not-digested and digested L-HK-BC17 oil are also shown; #, *p* < 0.05.

The L-HK-BC17, L-HK-BC17 oil suspension (10^7^ heat-killed cells/mL) and sunflower oil alone were also tested for antimicrobial and anti-biofilm activity toward pathogenic species, in order to exclude any undesired stimulating effect. The results reported in Figure 7A show that the L-HK-BC17 and L-HK-BC17 oil suspension reduced planktonic growth by 40–73% and 35–65%, respectively, in accordance with what was previously observed for the not-formulated HK-BC17. Similarly, the L-HK-BC17 and L-HK-BC17 oil suspension displayed antagonist activity toward biofilm formation and pre-formed biofilms of all tested pathogens (Figure 7B,C). Specifically, inhibition rates of 27–50% and eradication rates of 30–48% were found. No significant differences between the L-HK-BC17 and L-HK-BC17 oil suspension activity were observed (*p* > 0.05); sunflower oil did not interfere with planktonic and adherent growth of pathogens.

In addition, considering that the gastric half-emptying time is 48 ± 15 min and 78 ± 14 min in breastfed and formula-fed infants, respectively [29], the L-HK-BC17 oil suspension was pre-digested in simulated gastric fluid for 2 h to investigate the impact of the passage through the acidic gastric environment on the bifidogenic and antipathogenic effect. Although the effect slightly decreased compared to the not-digested formulation, the L-HK-BC17 oil suspension was still able to significantly enhance the planktonic growth of bifidobacteria (130–179%, Figure 5), the formation of biofilms (151–206%, Figure 6A), and pre-formed biofilms (128–182%, Figure 6B). At the same time, the pre-digested L-HK-BC17 oil suspension still reduced pathogenic planktonic growth (27–58%) and biofilms (21–37% inhibition, 22–35% eradication), although to a lesser extend with respect to the L-HK-BC17 oil suspension (Figure 7A–C).

**Figure 8:**
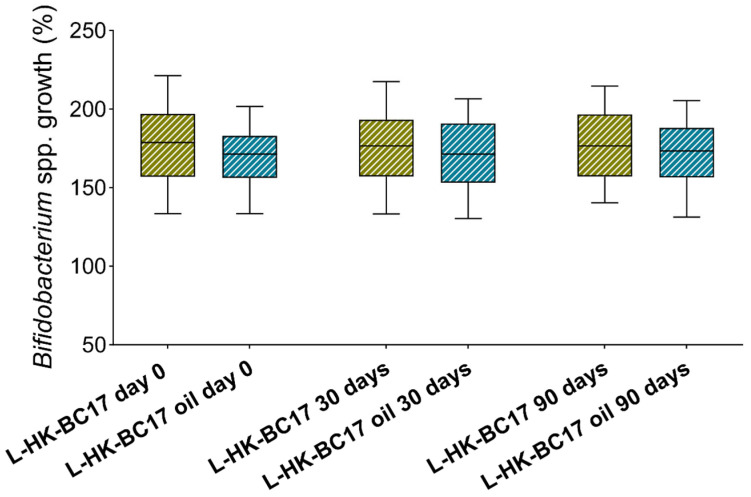
Effects of L-HK-BC17 and L-HK-BC17 oil on planktonic cultures of *Bifidobacterium* spp. over time. The effects of L-HK-BC17 and L-HK-BC17 oil were tested at 10^7^ heat-killed cells/mL immediately after preparation (day 0) and after 30 days and 90 days of storage. Each box represents the interquartile range (25th and 75th percentiles), and lines within the boxes indicate the median values for the samples. The extremes of the bars indicate the minimum and maximum values, respectively.

Finally, the bifidogenic activity of the L-HK-BC17 and L-HK-BC17 oil suspension was totally retained after 30 and 90 days of storage at +4–8 °C (*p* > 0.05) (Figure 8), indicating that HK-BC17 can be stored for a prolonged period both as lyophilized powder and oil suspension.

## 4. Discussion

The consumption of probiotics, prebiotics, or symbiotics during lactation can positively contribute to the colonization of the gut tract in newborns [30]. Several studies have demonstrated that the oral intake of *Bifidobacterium* spp. during the first 6 months of age leads to an increase in the number of gut bifidobacteria, with this effect being more pronounced in infants born by C-section [31,32,33]. In addition to bifidobacteria, lactobacilli have shown the ability to relieve the symptoms of gastrointestinal disorders, abdominal pain, diarrhea, and constipation in adults and children [34,35]. As an example, it has been shown that the supplementation of infant formula with *L. reuteri* DSM17938 promoted the growth of lactobacilli and modulated the early development of gut microbiota in C-section delivered newborns, but not in those born via vaginal delivery [36]. Lactobacilli are commonly isolated from fermented foods, the human gut, or breast milk, but they are also major components of a healthy human vaginal microbiota [37]. Although naturally born infants come in contact with maternal microorganisms during the passage through the birth canal, the impact of lactobacilli from vaginal niche on gut microbiota is still poorly elucidated.

In this regard, we have previously demonstrated that the culture supernatants of vaginal lactobacilli, especially that of *L. vaginalis* BC17, can stimulate the growth of *Bifidobacterium* spp., likely due to the secretion of metabolites that can serve as substrates [18]. Instead, in the present work, we focused on *L. vaginalis* BC17 cells to further investigate the strain’s capability to drive a bifidogenic shift in infants’ gut. 

However, when designing and proposing of a probiotic product, evaluating the safety profile for human use is a priority. The main factor to consider for safety issue is the assessment of antibiotic resistance. Indeed, a large number of lactobacilli carrying one or more genes related to tetracycline, erythromycin, aminoglycosides, vancomycin, and β-lactam antibiotics resistances have been reported. Although MIC cut-off values are not standardized for lactobacilli, the MIC values obtained for *L. vaginalis* BC17 are below the cut-off values defined for *L. reuteri*, which belongs to the same genus [12,26]. 

Nonetheless, a phenotypic test alone is not exhaustive since it does not discriminate between intrinsic and extrinsic resistance and does not guarantee the absence of transferable resistance genes. According to EFSA guidelines, whole genome sequencing is a valid technique to check microorganisms intentionally used as food supplements [13]. For this reason, the genome of *L. vaginalis* BC17 was fully sequenced, and the absence of antibiotic resistance genes was confirmed. This aligns with the finding that *Limosilactobacillus* strains are generally antibiotic susceptible, although strains from food-producing animals (i.e., *L. reuteri* strains) are more prone to acquire antibiotic resistance [38]. Moreover, the genome analysis made it possible to exclude the presence of other virulence factors that have been associated with lactobacilli, mainly causing evasion from host defense mechanisms, platelet aggregation, tissue disruption, and host invasion [12]. 

To date, only four complete genomic sequences of *L. vaginalis* strains have been deposited (RefSeq assembly accessions: PRJNA880306, PRJNA566211, PRJNA930673, PRJNA872822). These genomes have sizes between 1.78–1.89 Mbp and G + C contents of 40.4–40.7%, consistent with the findings for *L. vaginalis* BC17. The whole genome sequencing of *L. vaginalis* BC17 and the accomplishment of safety requirements can contribute to a deeper understanding of the genetic features of this species, in the perspective of a revising of *Limosilactobacillus vaginalis* by the international authorities and the recognition of the QPS status. 

Nevertheless, considering the intended application of *L. vaginalis* BC17 as a supplement for infants with potentially compromised gut integrity, such as those born by C-section or fed with formula, additional safety concerns may arise from the use of live cells, due to the possible translocation of orally administered lactobacilli from the gut to the systemic circulation and subsequent bacteriemia [39]. Furthermore, live probiotics can interfere with the normal colonization of gut microbiota in neonates, potentially altering normal immune system development [40].

To overcome the aforementioned limitations, a postbiotic preparation based on *L. vaginalis* BC17 has been proposed as a considerably safer alternative to live cells. Inactivation of probiotics can be conducted using various methods (i.e., heat, chemicals, gamma or ultraviolet rays, sonication) [41]. Among them, heat treatment is widely applied as it allows for the retention of the beneficial properties exerted by live probiotics in the intestine [42]. In the present work, heat inactivation of *L. vaginalis* BC17 was achieved by combining heat treatments (2 h at 70 °C) with incubation periods at low temperature (24 h at 4 °C). The inactivation process was applied to three different bathes of *L. vaginalis* BC17 cultures, with highly reproducible results.

There is evidence that different probiotic strains can restore intestinal homeostasis in their heat-inactivated form [42]. Although the mechanisms of action of postbiotics are not well understood, favorable effects have been observed in in vitro experiments, animal models, and clinical trials [43,44]. For example, heat-killed *Lacticaseibacillus rhamnosus* GG was reported to down-regulate proinflammatory mediators (i.e., IL8 and TNFα) and increase anti-inflammatory mediators (i.e., IL10) in both Caco-2 cells [45] and animal models [46], with effects comparable to those of live strains. 

Heat treatment can lead to the release of bacterial cytoplasmatic content (such as DNA) and cell-wall components. The latter include EPS, teichoic and lipoteichoic acids, peptidoglycans, lipopolysaccharides, and S-layer proteins, which have been correlated with anti-inflammatory, immunomodulating and antibacterial activities [43,47]. 

A heat-killed multi-strain preparation (containing *Lactobacillus acidophilus*, *Lactiplantibacillus plantarum*, *Limosilactobacillus fermentum* and *Enterococcus faecium*) reduced *Salmonella* invasion and induced inflammation in a mouse model, and the effects were attributed to EPS and lipoteichoic acid [48]. Antibacterial activities by heat-killed lactobacilli were also observed against diarrheagenic *E. coli*, *Campylobacter,* and *Helicobacter pylori* [16].

Consistent with these observations, HK-BC17 was found to reduce the proliferation of an opportunistic (i.e., *E. coli* SO107) and virulent gastrointestinal pathogens (i.e., ETEC, *S. enterica* and *Y. enterocolitica*) with inhibition percentages up to 58%. We also highlighted anti-biofilm activity against the same pathogens: a significant reduction in biofilm formation and dispersion of pre-formed biofilms treated with HK-BC17 were observed applying two complementary methods, namely CV staining and MTT assay, with similar results. This is particularly important as enteropathogens physiologically form robust biofilms on the gastrointestinal mucosa, making infections more challenging to eradicate and increasing the risk of chronicity [28]. 

The effects of heat-killed probiotics on bifidobacteria are still poorly understood. In this context, Warda and colleagues demonstrated that the commercial product Lactobacillus LB, a heat-treated ferment generated by two lactic acid bacteria (*L. fermentum* and *Lactobacillus delbrueckii*), stimulated the growth of a range of *Bifidobacterium* spp. in a fecal gut fermentation model [49]. Nonetheless, Lactobacillus LB preparation also contains lactose, which can promote the growth of lactose-utilizing bacteria, including bifidobacteria. To the best of our knowledge, we reported for the first time that a heat-killed lactic acid bacterium deriving from the vaginal niche (*L. vaginalis* BC17) can trigger a bifidogenic effect. This effect is likely attributable to the presence of HK-BC17 itself, as the cells were resuspended in saline solution, without adding other potential prebiotic components.

The bifidogenic activity was proved on several bifidobacterial species highly present in the gut microenvironment such as *B. breve*, *B. bifidum*, *B. longum*, *B. infantis*, *B. adolescentis,* and *B. angulatum* [27,50]. Bifidobacterial strains revealed different susceptibility to the stimulating action of HK-BC17, in accordance with what observed by Warda et al. [49].

It is important to highlight that, although the bifidogenic activity was dose-dependent, a stimulating capability was retained even at a concentration of HK-BC17 as low as 10^5^ heat-killed cells/mL, suggesting that a few heat-inactivated cells are sufficient to elicit the effects. 

In natural environments, the microbial biomass is predominantly found in the form of biofilm. The formation of biofilms by commensal *Bifidobacterium* spp. is essential for ensuring colonization of the infant gut tract and preventing the adhesion of pathogens [51]. We found out that HK-BC17 had a high capability to promote the formation of *Bifidobacterium* spp. biofilms and to improve already established biofilms. This suggests that supplementation with HK-BC17 in newborns can be useful both as an early intervention, when the biofilm has not yet established, and as maintenance therapy in the first months of life to support the permanence of *Bifidobacterium* spp. in the gut.

Although the results are very promising, in vitro models suffer some limitations due to the impossibility to simulate the complex interactions between microbiota, intestinal epithelium, and immune system cells that occur in vivo. To date, we have no indications about the mechanisms of action of HK-BC17. Warda et al. showed up that the dialysis of Lactobacillus LB attenuated its bifidogenic ability, thus proposing that the effects can be ascribed to some soluble cell membrane components [49]. In this regard, bifidogenic activity has been observed for EPS synthetized by different species of lactobacilli, including *Fructilactobacillus sanfranciscensis* [52], *L. plantarum* [53], and *L. rhamnosus* [54], but further investigations are required. Since genes related to the EPS synthesis pathway have been found in *L. vaginalis* BC17 genome, it can be speculated that EPS molecules contribute to the observed bifidogenic behavior.

It should be mentioned that heat-killed preparations offer also some pharmaceutical advantages over their live counterparts, since they are easier to standardize, transport, and store. Medical devices containing heat-inactivated strains, particularly *L. acidophilus*, *L. plantarum*, *L. rhamnosus,* and *Lactocaseibacillus casei*, have been commercialized for the treatment of colic, diarrhea, and gut dysbiosis in children and adults [16].

We proposed the inclusion of lyophilized HK-BC17 (L-HK-BC17) in an oil formulation aimed at the oral supplementation of C-delivered and formula-fed infants. In particular, the lyophilization of HK-BC17 made it possible to obtain a powder with high cell density (10^12^ cells/g) that was subsequently dispersed in sunflower oil at a final concentration of 10^10^ heat-killed cells/g of oil. Sunflower oil was chosen as a safe excipient, suitable for infant oral preparations. An estimate dose is about 0.1 g of oil suspension (five drops), equivalent to 10^9^ heat-killed cells. This dosage is in line with 10^8^–10^9^ cells/day recommended for probiotic formulations [14,55] and is sufficient to exert a biological effect in in vivo models by heat-inactivated cells [46,56]. 

The L-HK-BC17 oil suspension revealed favorable technological properties. Specifically, the sediment based on *L. vaginalis* BC17 powder can be easily re-dispersible upon shaking, as demonstrated by the low number of inversions needed for obtaining the complete re-dispersibility. This, in turn, assured the withdrawal of uniform doses and high stability [57]. Indeed, the preparation was physically stable and maintained its aesthetic appearance (color and odor) up to 3 months of storage. Moreover, the low viscosity of the formulation could allow an easy outpour from the orifice of the bottle or facilitate the manufacture and packaging processes [58].

Importantly, the formulation strategy was suitable to preserve the biological activity of HK-BC17, as the L-HK-BC17 and L-HK-BC17 oil suspension retained the bifidogenic activities toward planktonic cultures and biofilms of *Bifidobacterium* spp. observed for the not-formulated HK-BC17, along with the antipathogenic effect. On the contrary, sunflower oil itself did not affect bifidobacterial and pathogens growth. Finally, the bifidogenic activity did not decrease over the storage period and was almost preserved after digestion in simulated gastric fluid, with the latter characteristic being crucial for formulations designed for oral intake. We are aware that our data suffer from in vitro experimental limitations, and further investigations using microbial communities, gut-simulated models, or in vivo models are needed to translate the results in complex systems with the ultimate aim of proposing the HK-BC17 oil suspension as an effective agent for the sustainment of bifidobacteria proliferation in the infant gut tract.

## 5. Conclusions

*Limosilactobacillus vaginalis* BC17 heat-killed cells proved to be active in stimulating bifibacteria and counteracting pathogens growth, and a functional formulation for infant oral intake was developed. The present research deepens the potential of postbiotic agents in the perspective of infant microbiota shaping, without neglecting important safety issues.

## 6. Patents

All the authors are inventors of the following patent applications: “Ceppo di Lattobacillo Utilizzabile per Stimolare e Riequilibrare il Microbiota Intestinale” (nr. 102021000026339-ITALY), “*Lactobacillus* strain usable for stimulating and rebalancing intestinal microbiota” (nr. PCT/IB2022/059819) owned by Alma Mater Studiorum, University of Bologna, Italy.

## Figures and Tables

**Table 1:** Susceptibility of *L. vaginalis* BC17 to antibiotics expressed as MIC values (μg/mL). Microbiological cut-off values defined for *Limosilactobacillus reuteri* were also reported for comparison; they discriminate between resistant and susceptible strains and were taken from [26].

Antibiotic	MIC for *L. vaginalis* BC17	Microbiological Cut-Off Values for *L. reuteri*
Ampicillin	0.125	2
Clindamycin	<0.06	1
Tetracycline	1	16
Vancomycin	>256	n.r.
Gentamycin	0.25	8
Erythromycin	<0.03	1
Kanamycin	15	64
Streptomycin	7.5	64

n.r.: not required.

## Data Availability

Genome sequence of *L. vaginalis* BC17 is available at GenBank database under accession number CP122957.
